# Development of Small RNA Delivery Systems for Lung Cancer Therapy

**DOI:** 10.3390/ijms16035254

**Published:** 2015-03-06

**Authors:** Yu Fujita, Kazuyoshi Kuwano, Takahiro Ochiya

**Affiliations:** 1 Division of Molecular and Cellular Medicine, National Cancer Center Research Institute, Tokyo 104-0045, Japan; E-Mail: yufujit2@ncc.go.jp; 2 Division of Respiratory Diseases, Department of Internal Medicine, Jikei University School of Medicine, Tokyo 105-8461, Japan; E-Mail: kkuwano@jikei.ac.jp

**Keywords:** RNAi, siRNA, miRNA, drug delivery system, lung cancer

## Abstract

RNA interference (RNAi) has emerged as a powerful tool for studying target identification and holds promise for the development of therapeutic gene silencing. Recent advances in RNAi delivery and target selection provide remarkable opportunities for translational medical research. The induction of RNAi relies on small silencing RNAs, which affect specific messenger RNA (mRNA) degradation. Two types of small RNA molecules, small interfering RNAs (siRNAs) and microRNAs (miRNAs), have a central function in RNAi technology. The success of RNAi-based therapeutic delivery may be dependent upon uncovering a delivery route, sophisticated delivery carriers, and nucleic acid modifications. Lung cancer is still the leading cause of cancer death worldwide, for which novel therapeutic strategies are critically needed. Recently, we have reported a novel platform (PnkRNA™ and nkRNA^®^) to promote naked RNAi approaches through inhalation without delivery vehicles in lung cancer xenograft models. We suggest that a new class of RNAi therapeutic agent and local drug delivery system could also offer a promising RNAi-based strategy for clinical applications in cancer therapy. In this article, we show recent strategies for an RNAi delivery system and suggest the possible clinical usefulness of RNAi-based therapeutics for lung cancer treatment.

## 1. Introduction

RNA interference (RNAi) is one of the most exciting discoveries in molecular biology and represents a significant breakthrough in our understanding of the function and regulation of genes in cells [1]. Fire and Mello, who discovered it, were awarded the Nobel Prize for Medicine in 2006 [2]. The silencing technology to suppress the expression of various genes by using different molecules, such as small interfering RNA (siRNA), short hairpin RNA (shRNA), and microRNA (miRNA), is applicable to many kinds of therapeutics for human diseases caused by specific genes. They are all commonly cleaved by an endogenous enzyme called Dicer that gives rise to the important transcription factors involved in the gene expression regulation. RNAi is initiated by exposing cells to long, double-stranded (ds) RNAs via transfection or endogenous expression. The dsRNAs are processed into smaller fragments (usually 21–23 nucleotides in length) of siRNA [3], which form a complex with the RNA-induced silencing complexes (RISCs) [4]. The sense strand is then cleaved by an endonuclease of the RISC, the Argonaute 2 (AGO2), whereas the antisense strand guides the RISC toward the perfectly complementary target mRNA, which is further cleaved by AGO2 into two messenger RNA (mRNA) fragments [5,6]. Introduction of siRNA into cells leads to downregulation of target genes without triggering interferon responses [3]. The silencing technology to suppress the gene expression by using siRNAs is applicable to many kinds of research or therapeutics for human diseases caused by specific genes, which are difficult to regulate through traditional approaches. In the last 10 years, a remarkable effort has been made in therapeutic application of gene silencing in the human body. Indeed, phase I studies of siRNAs for the treatment of age-related macular degeneration and respiratory syncytial virus provided promising evidence without nonspecific toxicity [7,8]. The critical hurdles impeding the development of siRNAs are effective delivery to target sites, therapeutic potency, and elimination of off-target effects [9]. Despite the reliable potential of siRNAs, there are still technical problems to clear before clinical use, including safety, stability, and successful delivery of siRNAs to the appropriate tissue and into the appropriate cells. Additionally, the therapeutic technology to avoid undesirable innate immune responses, instability of nucleic acid, and off-target effects notably reduces the efficacy of RNAi molecules [10,11]. Therefore, the development of RNA modifications and sophisticated drug delivery systems (DDSs) for RNAi-based therapeutic strategies that are safer, more stable, and more effective is a main consideration. MiRNAs are short (usually 19–23 nucleotides in length), non-coding RNAs found in multiple organisms that regulate gene expression primarily by decreasing the levels of their target mRNAs through binding to specific target sites in the 3'-untranslated regions (3'-UTRs) of these mRNAs [12]. Most miRNAs are derived from primary miRNA transcripts containing a cap and a poly (A) tail that are produced by RNA polymerase II from the miRNA genes. The primary miRNAs are further cleaved into 22-nt mature miRNAs by the consecutive functions of RNAase III Drosha-DGCR8 and Dicer, which are present in the nucleus and cytoplasm, respectively. In animals, single-stranded miRNAs are assembled into an RISC and primarily bind target mRNAs at specific sequence motifs predominantly found in the 3'-UTRs of the transcripts. miRNAs are key players in various critical cellular processes, such as proliferation, cell cycle progression, apoptosis, and differentiation. As a consequence, aberrant expression of miRNAs is frequently observed in many diseases, including cancer [13]. Multiple instances of miRNA dysregulation have been investigated in various types of cancers over the past several years. Growing evidence has revealed that many miRNAs are upregulated or downregulated in various tumors. These miRNAs act either as oncogenes (“oncomiRs”) or tumor suppressors (“tumor suppressor miRNAs”) [14]. Compared to siRNA-based therapies, which are already in clinical trials, miRNAs have the potential to target multiple genes, potentially providing simultaneous regulation of the genes involved in various oncogenic pathways [15]. The success of miRNA-based therapeutic delivery is also dependent upon uncovering a delivery route that yields efficient outcomes, is convenient, and promotes patient compliance.

Oncology is one of the core areas that can benefit the most from this novel therapeutic strategy, as it allows for modulating the expression of any gene involved in tumor initiation, growth, and metastasis formation. Lung cancer is the most common cause of cancer-related death worldwide [16]. It is a heterogeneous disease with two predominant pathological types, non-small cell lung cancer (NSCLC) and small cell lung cancer (SCLC). About 85% of all lung cancers are categorized as NSCLC. NSCLC is divided into adenocarcinoma, squamous cell carcinoma, and large cell carcinoma. Various therapeutic strategies, including surgery, radiotherapy, chemotherapy, and molecular targeted therapies, are commonly used to treat NSCLC, either alone or in combination. Despite the development of novel molecular targeted strategies [17], the overall prognosis remains poor. Patients who initially respond to treatment often relapse and succumb to therapy-resistant tumors [18]. Therefore, novel therapeutic strategies that specifically target lung cancer cells are needed. In general, the delivery of RNAi-based therapeutics can be achieved through systemic administration or local administration. The systemic delivery may induce adverse events, such as aggregation and complement activation, liver toxicity and stimulation of the immune response [19]. For these reasons, we consider that local administration of RNAi-based therapeutics to the target cancer cells may be a promising approach to overcome the problems of systemic administration. Currently, we have reported a novel naked RNAi platform technology through inhalation without delivery vehicles in lung cancer xenograft models [20]. This delivery approach may be a successful strategy for lung cancer treatment using a novel stable RNAi platform with local delivery. In this review, we discuss recent advances in small RNA delivery strategies and the application of these systems in *in vivo* models and clinical trials for lung cancer therapy. Furthermore, we offer perspectives on future applications of siRNA and miRNA therapeutics and discuss the promise and limitations of delivery strategies for lung cancer.

## 2. The Development of siRNA-Based Therapeutics for Lung Cancer Treatment

The clinical application of RNAi-based therapeutics using siRNAs has been growing as the RNAi technology and platform have matured. Many siRNA-based therapeutics are being assessed in preclinical and clinical trials, and this research provides further opportunities for successful results [21]. Indeed, there are some drug candidates for clinical development in 2015. Remarkably, there are a number of sites for local administration, such as the skin, retina, and lungs, which permit safe and efficient delivery without side effects. For example, the siRNA therapeutic, ALN-RSV01, is directed against the mRNA encoding the N protein of the respiratory syncytial virus (RSV) that exhibits specific anti-RSV activity. Presently, phase II clinical trials have been initiated for the treatment of RSV infection, using intranasally naked siRNA molecules [8,22]. Although the mechanism of how naked siRNAs can entry into cells to initiate RNAi is unclear, the lungs and eyes are two of the very few organs in the body where successful RNAi could be achieved by local delivery of naked siRNAs. Some drug companies working in RNAi therapy are chemically modifying their oligonucleotides. These siRNAs are modified with 2'-*O*-methylation to protect against nuclease degradation and protect an innate immune response [23]. The successful approach for clinical application may be to combine modified siRNA with local administration. For this reason, direct administration of RNAi-based therapeutics to target organs is a promising approach to overcoming some problems of systemic administration. Local delivery offers a new method for the treatment of various human diseases [24]. Indeed, systemic administration of siRNAs is still the most widely used administration route in preclinical studies. Currently, six cancer clinical trials are underway using nanoparticle-based siRNA delivery, evaluating the initial safety and utility of these treatments (Table 1).

**Table 1:** Small interfering RNA (siRNA)-based therapeutics for cancer treatment in clinical trials.

Drug	Target Gene	Delivery Methods	Disease	Vehicle	Phase	Year
CALAA-01	*RRM2*	Intravenous injection	Solid tumors	Cyclodextrin nanoparticle	I	2008
TKM 080301	*PLK1*	Intravenous injection	Solid tumors with liver involvement	Lipid nanoparticle (LNP)	I/II	2010
ALN-VSP02	*KSP/VEGF*	Intravenous injection	Solid tumors with liver involvement	Lipid nanoparticle (LNP)	I	2009
Atu027	*PKN3*	Intravenous injection	Solid tumors	Lipid nanoparticle (LNP)	I	2009
siG12D LODER	*KRAS-G12D*	EUS biopsy needle	Pancreatic ductal adenocarcinoma	LODER polymer	II	2011
siRNA-EphA2-DOPC	*EphA2*	Intravenous injection	Solid tumors	DOPC	I	2012

The first siRNA phase I trial, CALLA-01, was developed by several pharmaceutical companies for solid tumors; it targets the M2 subunit of ribonucleotide reductase (RRM2) to inhibit tumor growth [25]. Additionally, siRNAs targeting polo-like kinase 1 (PLK1), the kinesin spindle protein (KSP), the vascular endothelial growth factor (VEGF), and protein kinase N3 (PKN3), which are formulated with lipid nanoparticle, have been developed as candidate pipelines in the phase I trial. Silenseed, Ltd., initiated a phase II trial to estimate the survival of patients treated with siG12D local drug eluter (LODER). The siG12D LODER is a polymeric matrix that encompasses the siRNA target to the mutated v-Ki-ras2 Kirsten rat sarcoma viral oncogene homolog (KRAS) oncogene, designed to release the drug directly within a pancreatic ductal adenocarcinoma. More than 90% of pancreatic ductal adenocarcinomas involve mutations in the KRAS oncogene, with the most common being G12D. The siG12D LODER is placed in the tumor using a biopsy needle during an endoscopic ultrasound (EUS) biopsy procedure. In an upcoming phase II study, a single dose of siG12D LODER will be administered to patients with advanced pancreatic cancer combined with cytotoxic chemotherapy. Lastly, Landen *et al.* have developed a 1,2-dioleoyl-*sn*-glycero-3-phosphocholine (DOPC)-based nanoparticle with siRNA targeted to EphA2, a tyrosine kinase receptor highly overexpressed in human cancers [26]. Currently, the M.D. Anderson Cancer Center has initiated a phase I trial to estimate the safety of siRNA-EphA2-DOPC.

Lung cancer is one of the most common tumors worldwide with regard to incidence rates and mortality. Various therapeutic target genes for lung cancer therapy have already been identified. So far, there have already been significant improvements in siRNAs for primary or metastatic lung cancer *in vivo* models by targeting various types of genes, such as ribophorin II (RPN2) [20], chromosome 7 open reading frame 24 (C7orf24) [27], myeloid cell leukemia sequence 1 (Mcl1) [28], CD31 [29], insulin-like growth factor receptor 1 (IGF-1R) [30], survivin [31,32,33], multidrug resistance-associated protein 1 (MRP1) [34,35], luciferase [36,37], bcl-2 [35,38], v-akt murine thymoma viral oncogene homolog 1 (Akt1) [39,40], sodium-dependent phosphate co-transporter 2b (NPT2b) [41], mouse double minute 2 (MDM2) [42,43], signal transducer and activator of transcription 3 (STAT3) [44], v-myc avian myelocytomatosis viral oncogene homolog (c-myc) [43,45], and VEGF [43,46] (Table 2). These data suggest that siRNA-based therapeutics have potential for a reliable strategy against lung cancer. In lung cancer treatment using siRNA-based therapeutics, systemic administration as well as local administration can be exploited to successfully deliver treatment to the lungs. Some of these studies have successfully shown the efficacy of RNAi-based therapy through intrapulmonary administration of siRNAs. A local and less invasive delivery route for easily accessible administration of siRNA may provide the therapeutic advantage in lung cancer treatment. The administration route may have to be carefully chosen based on the therapeutic application. In addition, non-viral carriers, such as lipids, polymer nanoparticles, and inorganic compounds, offer the advantages of chemical modifications and tailoring to the needs of sophisticated siRNA delivery *in vivo* [47,48]. Lipid-based and polymer-based nanoparticles can reduce the negative electrical charge of RNA nucleotides to promote cell uptake [49]. Indeed, viral vectors, such as adenoviral [50,51] or lentiviral vectors [52,53,54,55], can be still used to transfer siRNAs to lung cancer cells. Although their safety regarding toxicity and immunogenicity are challenges for clinical applications, these nanocarriers are needed to efficiently deliver siRNA-based therapeutics. Some recent studies have described intrapulmonary administration of naked nucleic acids for siRNAs in the lungs [20,27]. We suggest that successful delivery of RNAi-based therapeutics requires patient compliance with the intended delivery route and efficient delivery carriers.

**Table 2:** siRNA-based therapeutics for lung cancer treatment in *in vivo* studies.

Target Gene	Administration	Type of siRNA Delivery	References
*RPN2*	Intratracheal	Naked nucleic acids	[20]
*C7orf24*	Intratumoral	Naked nucleic acids	[27]
*Mcl1*	Intratracheal	Ethylphosphocholine-based lipoplexes	[28]
*CD31*	Intravenous	AtuFECT01 lipoplexes	[29]
*IGF-1R*	Intravenous	Magnetic lipoplexes	[56]
*Survivin*	Intravenous	Liposomes	[31]
	Intravenous	Polyethylenimine (PEI)	[32,33]
*MRP1*	Inhalation	Liposomes	[34]
*Luciferase*	Inhalation	Chitosan	[36]
	Intravenous	Lipid/Calcium/Phosphate (LCP)	[37]
*Bcl-2*	Intravenous	Cationic bovine serum albumin	[38]
*Akt1*	Inhalation	Glycerol propoxylate triacrylate-spermine	[39,40]
*NPT2b*	Inhalation	Glycerol propoxylate triacrylate-spermine	[41]
*MDM2*	Intravenous	Poly(methacryloyloxy ethyl phosphorylcholine)-block-poly(diisopropanolamine ethyl methacrylate) (PDMA-b-PDPA)	[42]
*STAT3*	Intraperitoneal	PEI and poly-l-lactic-co-glycolic acid (PLGA)	[44]
*MDM2, c-myc, VEGF*	Intravenous	LCP	[43]
*VEGF*	Intravenous	LCP	[46]
*c-Myc*	Intratracheal	Arginine-glycine-aspartic acid (RGD) gold nanoparticles	[45]
*MRP1 and Bcl-2*	Inhalation	Lutein hormone releasing hormone (LHRH)-modified mesoporous silica nanoparticles (MSN)	[35]

## 3. The Development of microRNA-Based Therapeutics for Lung Cancer Treatment

Recently, miRNAs were shown to upregulate target-gene expression by either directly binding to the target mRNA or indirectly repressing nonsense-mediated RNA decay [57,58]. The development of miRNA-based therapeutics represents a novel strategy for cancer treatment and is growing rapidly with the help of RNAi technologies. For therapeutic applications of miRNA, the miRNA expression levels in cancer cells have to be artificially controlled. Compared to the siRNA-based therapies that are already in clinical studies, miRNAs are low in toxicity and have the potential to simultaneously target multiple genes that cooperate in the same pathway [15]. As presented above, miRNAs are generally classified as oncomiRs or tumor-suppressor miRNAs, with different therapeutic approaches developed for each miRNA class. In general, the upregulation of miRNA expression is achieved through administration of synthetic miRNA mimics or miRNA-expressing vectors. The miRNA replacement therapy involves the re-introduction of a tumor suppressor miRNA to reverse the loss of miRNA function. On the other hand, the downregulation of miRNA expression is achieved through administration of antisense nucleotides, often chemically modified to improve thermal stability and specificity. The chemical modifications, such as the 2'-*O*-methyl-group and locked nucleic acid (LNA), can improve miRNA stability and affinity [59]. Currently, oncomiRs and tumor-suppressive miRNAs in various types of cancer have been identified, and a number of miRNA-based preclinical studies have been conducted by various companies, such as Regulus Therapeutics, miRagen Therapeutics, Sanofi, and Mirna Therapeutics (Table 3). Indeed, Mirna Therapeutics, Inc., has initiated clinical trials for miR-34 (MRX34) against hepatocellular carcinoma, making this one of the first miRNA replacement therapies to enter clinical trials [60]. The phase I study of MRX34 will be completed at the end of the first quarter of 2015. Remarkably, a preclinical trial in lung cancer patients using a *let-7* replacement therapy has been initiated by Mirna Therapeutics. Many studies have already shown that *let-7* family miRNAs act as key tumor suppressors in regulating cell survival and proliferation in lung cancers [61,62,63,64,65,66]. Esquela-Kercher *et al.* and Trang *et al.* have reported that intranasal administration of a *let-7* mimic into lung cancer xenograft models significantly reduced tumor growth [67,68]. These data suggest that *let-7* replacement therapy is indeed a promising therapeutic treatment for humans.

**Table 3:** miRNA-based therapeutics for cancer treatment in development.

microRNA	Modulation Strategy	Diseases	Status	Company
miR-10b	Inhibition	Glioblastoma	Preclinical	Regulus Therapeutics
miR-21	Inhibition	Hepatocellular carcinoma	Preclinical	Regulus Therapeutics
miR-155	Inhibition	Hematological malignancies	Preclinical	miRagen Therapeutics
miR-221	Inhibition	Hepatocellular carcinoma	Preclinical	Sanofi
*let-7*	Replacement	Lung cancer	Preclinical	Mirna Therapeutics
miR-16	Replacement	Cancer	Preclinical	Mirna Therapeutics
miR-34	Replacement	Hepatocellular carcinoma	Phase I	Mirna Therapeutics

In addition to the *let-7* family, there have been some potential therapeutic miRNAs for lung cancer treatment *in vivo*, including miR-7 [69], miR-29b [70], miR-34a [71], miR-145 [72], miR-150 [73], and miR-200c [74] (Table 4). Some of these studies have shown that new delivery technologies using non-viral carriers, such as polyethylenimine and NOV340 liposomes, have the potential for an appropriate delivery of miRNAs for lung cancer treatment. These non-viral delivery systems could represent a genetically and immunogenically safe technology for miRNA-based therapies [75]. Delivery of these candidate miRNAs as synthetic miRNA mimics or miRNA inhibitor will be a promising therapeutic approach to treat lung cancer in clinical trials.

**Table 4:** miRNA-based therapeutic strategies for *in vivo* models of lung cancer.

miRNA	Administration	Modulation Strategy	Delivery Technology	References
*let-7*	Intranasal	Replacement	Adenoviruses	[67]
	Intravenous	Replacement	Neutral liposomes	[61]
	Intratracheal	Replacement	Lentiviruses	[65]
miR-7	Intratumoral	Replacement	Cationic liposomes	[69]
miR-29b	Intravenous	Replacement	Cationic liposomes	[70]
miR-34a	Intratumoral	Replacement	Neutral liposomes	[71]
miR-145	Intratumoral	Replacement	PEI	[72]
miR-150	Intratumoral	Inhibition	Cationic liposomes	[73]
miR-200c	Intravenous	Replacement	Liposomes (NOV340)	[74]

## 4. A Novel RNAi Platform for Lung Cancer Treatment 

Several RNAi-based therapeutics are being assessed in preclinical and clinical trials, and these studies provide further opportunities for successful results [21,76]. We consider that the success of RNAi-based therapeutic delivery is dependent upon uncovering a delivery route and sophisticated delivery carriers that yield efficient outcomes and safety.

Firstly, the lung is anatomically accessible to therapeutic drugs via the pulmonary route. Accessibility is a key requirement for successful RNAi-based *in vivo* and clinical studies, and this anatomical characteristic offers several important benefits over systemic delivery, including the use of lower doses of siRNAs and miRNAs, the reduction of undesirable systemic side effects, and improved stability due to reduced nuclease activity in the airways compared to serum [24]. The local approach could potentially enhance the retention of RNAi-based therapeutics in the lungs. Moreover, we understand that local delivery systems combined with nucleic acid modifications or sophisticated nanocarriers can more effectively enhance RNAi effects [77]. Secondly, an important point for RNAi-mediated silencing is whether the observed effects are specific rather than due to off-target effects and free from potential interferon responses [78,79]. Non-viral vectors, including lipid and polymer-based carriers, have been generally used for the delivery of RNAi-based therapeutics due to their reduced toxicity [80]. Non-viral vectors are safer, of low cost, and more reproducible. Toxicity results from characteristics of the packaging lipid or polymer, such as the length, saturation, or branching of polymer [81]. Efforts to reduce the toxicity of non-viral vectors have largely resulted in efforts to make the vectors more biodegradable and biocompatible. On the other hands, viral vector are the most effective, but their application is limited because of their immunogenicity, oncogenicity, and the small size of the DNA they can deliver. Although the non-viral delivery carriers elicit a relatively weak immune response than viral vectors, immune activation is triggered by systemic and local delivery of both lipoplexes and polyplexes [82,83]. The main limitation of non-viral delivery systems is their low transfection efficiency. Therefore, we consider that effective RNAi delivery to a local area in lungs requires more attention to the development of sophisticated non-toxic delivery vectors. Moreover, novel RNAi platform technologies to promote efficacy, the chemical stability and safety of the RNAi-therapeutics are needed.

**Figure 1:**
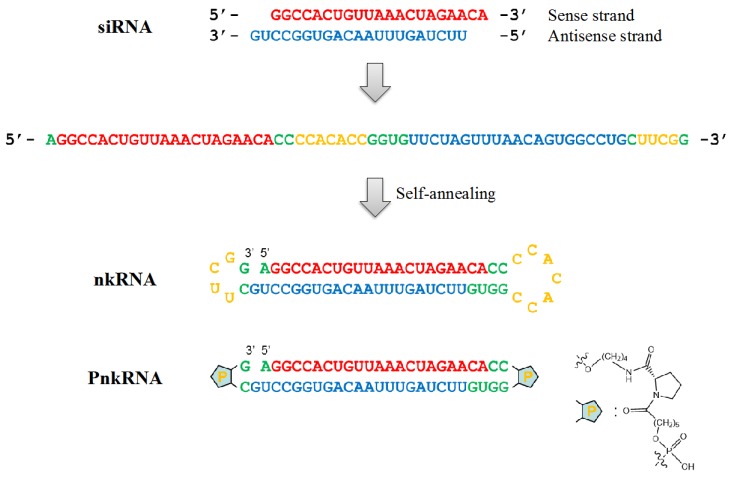
Shematic diagram of novel RNAi agents. Both nkRNA and PnkRNA were prepared as single-stranded RNA oligomers that then self-anneal. Nucleotides in red indicate the sense strand of the target (RPN2); nucleotides in blue indicate the antisense strand; and nucleotides in green and yellow indicate the loop cassettes. “P” indicates a proline derivative.

Here, we show a new RNAi platform technology with an inhalation approach without delivery carriers in lung cancer xenograft models (SCID mice). Hamasaki *et al.* have reported that a new class of RNAi agents, PnkRNA™ and nkRNA^®^, has a unique helical structure containing a central stem and two loops [84]. Large-scale production of these agents at low cost is possible because they do not require an annealing step (Figure 1). The novel RNAi platforms are more resistant to degradation than siRNA *in vitro* because of their unique structure [20,84]. The *in vitro* assay with labeled RNAi agents showed that these platforms were successfully incorporated into the cell cytoplasm by endocytosis [20]. The intrapulmonary delivery of novel RNAi agents was not associated with the expression of interferon (IFN)-α or IFN-β in the mouse model of lung diseases, suggesting that they might provide a solution to safety concerns about the off-target effects of canonical siRNAs [84]. Moreover, normal lung tissues have shown significant cytotoxicity from the RNAi treatment. We found that the novel RNAi therapeutic agents are more resistant to degradation, less immunogenic and less cytotoxic than siRNA, and capable of efficient intracellular delivery. On the basis of these promising evidences, we explored the effectiveness of this novel class of RNAi therapeutic agents against lung cancer [20]. To screen for target genes showing the growth inhibition of lung cancer cells, we selected ribophorin II (RPN2) as a target gene. Previously, Honma *et al.* showed that RPN2, which is part of an *N*-oligosaccharyl transferase complex, regulates the glycosylation of multi-drug resistance (MDR1) [85]. RPN2 siRNA decreased the membrane localization of P-glycoprotein by reducing its glycosylation status and restored the sensitivity to docetaxel. Recently, we have reported that RPN2 is involved in the regulation of lethal lung cancer phenotypes and represents a promising novel target for RNAi-based medicine against non-small cell lung cancer (NSCLC) [86]. In the *in vitro* assay, RPN2 siRNA and novel RNAi therapeutics inhibited A549-luc-C8 cell proliferation and suppressed RPN2 expression. Furthermore, we have demonstrated that the novel RNAi agent targeting RPN2 markedly suppressed the growth of A549-luc-C8 xenograft tumors (SCID mice) *in vivo.* Remarkably, we showed that the novel RNAi agent was administered in naked, unmodified form by aerosol. These data suggested that the local delivery of naked novel RNAi therapeutics could be a safe strategy for inhibiting lung cancer growth *in vivo*. This new technology using aerosol delivery can represent a safe, potentially RNAi-based strategy for clinical applications in lung cancer treatment without delivery vehicles [20]. As a limitation of this study, it is unclear whether naked novel RNAi-therapeutics would be successful in a host with an intact immune system. In fact, there are some physiological barriers in the lungs, such as the mucociliary clearance actions and phagocytosis by macrophages [24]. Furthermore, we have to pay careful attention to the behavioral difference between xenograft cancer mouse models and spontaneous lung tumor models. It is important to test the efficacy of naked-PnkRNA delivery technology in appropriate mice models. In any case, naked RNAi delivery has some advantages, such as simple formation and the absence of toxicity or inflammatory responses. This approach could open up new possibilities for treatment of various cancers and improve the clinical outcome for cancer patients.

## 5. Conclusions

**Figure 2:**
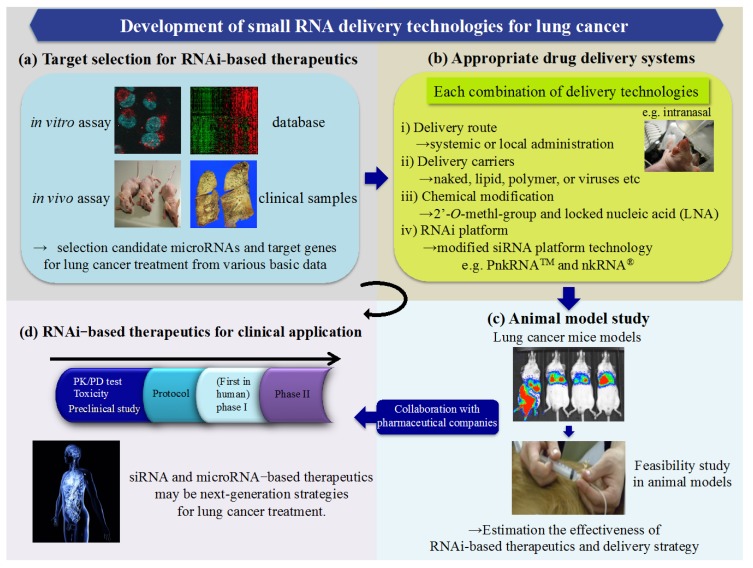
The development roadmap of RNAi-based therapeutics for lung cancer. This shows an outline of the development of small RNA delivery technologies for lung cancer. First, the success of an RNAi-based therapy rests on careful selection of target genes and miRNAs based on various basic data (**a**); Moreover, a delivery route, sophisticated delivery carriers, chemical modification, and modified RNAi platforms are needed to enhance RNAi effects in the cells of lungs. Appropriate drug delivery systems depend on each combination of these delivery technologies (**b**); We also need to estimate the effectiveness of RNAi-based therapeutics and delivery strategies in animal feasibility studies (**c**); Through collaboration with pharmaceutical companies, siRNA- and microRNA-based therapeutics will begin preclinical and clinical development (**d**). These therapeutics may be next-generation strategies for lung cancer treatment.

Development of RNAi-based therapeutics can provide novel opportunities for lung cancer treatment (Figure 2). The success of an RNAi-based therapy in clinical trials rests on careful selection of target genes and miRNAs. Moreover, we suggest that a delivery route, sophisticated delivery carriers, chemical modification, and modified RNAi platforms are needed to enhance RNAi effects in cancer cells. We also showed a unique RNAi delivery system using modified siRNA platform technology for lung cancer treatment. The establishment of small RNA delivery strategies opens the door to possible applications for lung cancer and several lung diseases.

## References

[1] Zamore P.D., Tuschl T., Sharp P.A., Bartel D.P. (2000). RNAi: Double-stranded RNA directs the ATP-dependent cleavage of mRNA at 21 to 23 nucleotide intervals. Cell.

[2] Fire A., Xu S., Montgomery M.K., Kostas S.A., Driver S.E., Mello C.C. (1998). Potent and specific genetic interference by double-stranded RNA in Caenorhabditis elegans. Nature.

[3] Elbashir S.M., Harborth J., Lendeckel W., Yalcin A., Weber K., Tuschl T. (2001). Duplexes of 21-nucleotide RNAs mediate RNA interference in cultured mammalian cells. Nature.

[4] Bernstein E., Caudy A.A., Hammond S.M., Hannon G.J. (2001). Role for a bidentate ribonuclease in the initiation step of RNA interference. Nature.

[5] Aagaard L., Rossi J.J. (2007). RNAi therapeutics: Principles, prospects and challenges. Adv. Drug Deliv. Rev..

[6] De Fougerolles A., Vornlocher H.P., Maraganore J., Lieberman J. (2007). Interfering with disease: A progress report on siRNA-based therapeutics. Nat. Rev. Drug Deliv..

[7] Check E. (2005). A crucial test. Nat. Med..

[8] DeVincenzo J., Cehelsky J.E., Alvarez R., Elbashir S., Harborth J., Toudjarska I., Nechev L., Murugaiah V., van Vliet A., Vaishnaw A.K. (2008). Evaluation of the safety, tolerability and pharmacokinetics of ALN-RSV01, a novel RNAi antiviral therapeutic directed against respiratory syncytial virus (RSV). Antivir. Res..

[9] Boudreau R.L., Martins I., Davidson B.L. (2009). Artificial microRNAs as siRNA shuttles: Improved safety as compared to shRNAs *in vitro* and *in vivo*. Mol. Ther..

[10] Burchard J., Jackson A.L., Malkov V., Needham R.H., Tan Y., Bartz S.R., Dai H., Sachs A.B., Linsley P.S. (2009). MicroRNA-like off-target transcript regulation by siRNAs is species specific. RNA.

[11] De Veer M.J., Sledz C.A., Williams B.R. (2005). Detection of foreign RNA: Implications for RNAi. Immunol. Cell Biol..

[12] Winter J., Jung S., Keller S., Gregory R.I., Diederichs S. (2009). Many roads to maturity: MicroRNA biogenesis pathways and their regulation. Nat. Cell Biol..

[13] Croce C.M. (2009). Causes and consequences of microRNA dysregulation in cancer. Nat. Rev. Genet..

[14] Esquela-Kerscher A., Slack F.J. (2006). Oncomirs—MicroRNAs with a role in cancer. Nat. Rev. Cancer.

[15] Pasquinelli A.E., Hunter S., Bracht J. (2005). MicroRNAs: A developing story. Curr. Opin. Genet. Dev..

[16] Ramalingam S.S., Owonikoko T.K., Khuri F.R. (2011). Lung cancer: New biological insights and recent therapeutic advances. CA Cancer J. Clin..

[17] Pao W., Girard N. (2011). New driver mutations in non-small-cell lung cancer. Lancet Oncol..

[18] Turrisi A.T., Sherman C.A. (2002). The treatment of limited small cell lung cancer: A report of the progress made and future prospects. Eur. J. Cancer.

[19] Kleinman M.E., Yamada K., Takeda A., Chandrasekaran V., Nozaki M., Baffi J.Z., Albuquerque R.J., Yamasaki S., Itaya M., Pan Y. (2008). Sequence- and target-independent angiogenesis suppression by siRNA via TLR3. Nature.

[20] Fujita Y., Takeshita F., Mizutani T., Ohgi T., Kuwano K., Ochiya T. (2013). A novel platform to enable inhaled naked RNAi medicine for lung cancer. Sci. Rep..

[21] Davidson B.L., McCray P.B. (2011). Current prospects for RNA interference-based therapies. Nat. Rev. Genet..

[22] DeVincenzo J., Lambkin-Williams R., Wilkinson T., Cehelsky J., Nochur S., Walsh E., Meyers R., Gollob J., Vaishnaw A. (2010). A randomized, double-blind, placebo-controlled study of an RNAi-based therapy directed against respiratory syncytial virus. Proc. Natl. Acad. Sci. USA.

[23] Bouchie A. (2012). Companies in footrace to deliver RNAi. Nat. Biotechnol..

[24] Fujita Y., Takeshita F., Kuwano K., Ochiya T. (2013). RNAi therapeutic platforms for lung diseases. Pharmaceuticals.

[25] Davis M.E., Zuckerman J.E., Choi C.H., Seligson D., Tolcher A., Alabi C.A., Yen Y., Heidel J.D., Ribas A. (2010). Evidence of RNAi in humans from systemically administered siRNA via targeted nanoparticles. Nature.

[26] Landen C.N., Chavez-Reyes A., Bucana C., Schmandt R., Deavers M.T., Lopez-Berestein G., Sood A.K. (2005). Therapeutic *EphA2* gene targeting *in vivo* using neutral liposomal small interfering RNA delivery. Cancer Res..

[27] Hama S., Arata M., Nakamura I., Kasetani T., Itakura S., Tsuchiya H., Yoshiki T., Kogure K. (2012). Prevention of tumor growth by needle-free jet injection of anti-C7orf24 siRNA. Cancer Gene Ther..

[28] Shim G., Choi H.W., Lee S., Choi J., Yu Y.H., Park D.E., Choi Y., Kim C.W., Oh Y.K. (2013). Enhanced intrapulmonary delivery of anticancer siRNA for lung cancer therapy using cationic ethylphosphocholine-based nanolipoplexes. Mol. Ther..

[29] Fehring V., Schaeper U., Ahrens K., Santel A., Keil O., Eisermann M., Giese K., Kaufmann J. (2014). Delivery of therapeutic siRNA to the lung endothelium via novel Lipoplex formulation DACC. Mol. Ther..

[30] Dong A.Q., Kong M.J., Ma Z.Y., Qian J.F., Xu X.H. (2007). Down-regulation of IGF-IR using small, interfering, hairpin RNA (siRNA) inhibits growth of human lung cancer cell line A549 *in vitro* and in nude mice. Cell Biol. Int..

[31] Tian H., Liu S., Zhang J., Zhang S., Cheng L., Li C., Zhang X., Dail L., Fan P., Dai L. (2012). Enhancement of cisplatin sensitivity in lung cancer xenografts by liposome-mediated delivery of the plasmid expressing small hairpin RNA targeting Survivin. J. Biomed. Nanotechnol..

[32] Kedinger V., Meulle A., Zounib O., Bonnet M.E., Gossart J.B., Benoit E., Messmer M., Shankaranarayanan P., Behr J.P., Erbacher P. (2013). Sticky siRNAs targeting survivin and cyclin B1 exert an antitumoral effect on melanoma subcutaneous xenografts and lung metastases. BMC Cancer.

[33] Bonnet M.E., Gossart J.B., Benoit E., Messmer M., Zounib O., Moreau V., Behr J.P., Lenne-Samuel N., Kedinger V., Meulle A. (2013). Systemic delivery of sticky siRNAs targeting the cell cycle for lung tumor metastasis inhibition. J. Control. Release.

[34] Mainelis G., Seshadri S., Garbuzenko O.B., Han T., Wang Z., Minko T. (2013). Characterization and application of a nose-only exposure chamber for inhalation delivery of liposomal drugs and nucleic acids to mice. J. Aerosol Med. Pulm. Drug Deliv..

[35] Taratula O., Garbuzenko O.B., Chen A.M., Minko T. (2011). Innovative strategy for treatment of lung cancer: Targeted nanotechnology-based inhalation co-delivery of anticancer drugs and siRNA. J. Drug Targets.

[36] Okuda T., Kito D., Oiwa A., Fukushima M., Hira D., Okamoto H. (2013). Gene silencing in a mouse lung metastasis model by an inhalable dry small interfering RNA powder prepared using the supercritical carbon dioxide technique. Biol. Pharm. Bull..

[37] Li J., Yang Y., Huang L. (2012). Calcium phosphate nanoparticles with an asymmetric lipid bilayer coating for siRNA delivery to the tumor. J. Control. Release.

[38] Han J., Wang Q., Zhang Z., Gong T., Sun X. (2014). Cationic bovine serum albumin based self-assembled nanoparticles as siRNA delivery vector for treating lung metastatic cancer. Small.

[39] Jiang H.L., Hong S.H., Kim Y.K., Islam M.A., Kim H.J., Choi Y.J., Nah J.W., Lee K.H., Han K.W., Chae C. (2011). Aerosol delivery of spermine-based poly(amino ester)/Akt1 shRNA complexes for lung cancer gene therapy. Int. J. Pharm..

[40] Hong S.H., Kim J.E., Kim Y.K., Minai-Tehrani A., Shin J.Y., Kang B., Kim H.J., Cho C.S., Chae C., Jiang H.L. (2012). Suppression of lung cancer progression by biocompatible glycerol triacrylate-spermine-mediated delivery of shAkt1. Int. J. Nanomed..

[41] Hong S.H., Minai-Tehrani A., Chang S.H., Jiang H.L., Lee S., Lee A.Y., Seo H.W., Chae C., Beck G.R., Cho M.H. (2013). Knockdown of the sodium-dependent phosphate co-transporter 2b (NPT2b) suppresses lung tumorigenesis. PLoS One.

[42] Yu H., Zou Y., Jiang L., Yin Q., He X., Chen L., Zhang Z., Gu W., Li Y. (2013). Induction of apoptosis in non-small cell lung cancer by downregulation of MDM2 using pH-responsive PMPC-b-PDPA/siRNA complex nanoparticles. Biomaterials.

[43] Yang Y., Li J., Liu F., Huang L. (2012). Systemic delivery of siRNA via LCP nanoparticle efficiently inhibits lung metastasis. Mol. Ther..

[44] Das J., Das S., Paul A., Samadder A., Bhattacharyya S.S., Khuda-Bukhsh A.R. (2014). Assessment of drug delivery and anticancer potentials of nanoparticles-loaded siRNA targeting STAT3 in lung cancer, *in vitro* and *in vivo*. Toxicol. Lett..

[45] Conde J., Tian F., Hernandez Y., Bao C., Cui D., Janssen K.P., Ibarra M.R., Baptista P.V., Stoeger T., de la Fuente J.M. (2013). *In vivo* tumor targeting via nanoparticle-mediated therapeutic siRNA coupled to inflammatory response in lung cancer mouse models. Biomaterials.

[46] Zhang Y., Schwerbrock N.M., Rogers A.B., Kim W.Y., Huang L. (2013). Codelivery of VEGF siRNA and gemcitabine monophosphate in a single nanoparticle formulation for effective treatment of NSCLC. Mol. Ther..

[47] Dehousse V., Garbacki N., Colige A., Evrard B. (2010). Development of pH-responsive nanocarriers using trimethylchitosans and methacrylic acid copolymer for siRNA delivery. Biomaterials.

[48] Fernandez C.A., Rice K.G. (2009). Engineered nanoscaled polyplex gene delivery systems. Mol. Pharm..

[49] Wu Y., Crawford M., Yu B., Mao Y., Nana-Sinkam S.P., Lee L.J. (2011). MicroRNA delivery by cationic lipoplexes for lung cancer therapy. Mol. Pharm..

[50] Xia L., Guan W., Wang D., Zhang Y.S., Zeng L.L., Li Z.P., Wang G., Yang Z.Z. (2013). Killing effect of Ad5/F35-APE1 siRNA recombinant adenovirus in combination with hematoporphrphyrin derivative-mediated photodynamic therapy on human nonsmall cell lung cancer. BioMed Res. Int..

[51] Gu X., Cun Y., Li M., Qing Y., Jin F., Zhong Z., Dai N., Qian C., Sui J., Wang D. (2013). Human apurinic/apyrimidinic endonuclease siRNA inhibits the angiogenesis induced by X-ray irradiation in lung cancer cells. Int. J. Med. Sci..

[52] Wang X.M., Cui J.W., Li W., Cai L., Song W., Wang G.J. (2012). Silencing of the *COPS3* gene by siRNA reduces proliferation of lung cancer cells most likely via induction of cell cycle arrest and apoptosis. Asian Pac. J. Cancer Prev..

[53] Lian Y.X., Chen R., Xu Y.H., Peng C.L., Hu H.C. (2012). Effect of protein-tyrosine phosphatase 4A3 by small interfering RNA on the proliferation of lung cancer. Gene.

[54] Liu Y., Yan X., Liu N., Zhou J., Liu J., Pang H., Cao J., Liu Y., Wang Y., Liu L. (2012). Lentivirus-delivered ZEB-1 small interfering RNA inhibits lung adenocarcinoma cell growth *in vitro* and *in vivo*. J. Cancer Res. Clin. Oncol..

[55] Caino M.C., Lopez-Haber C., Kim J., Mochly-Rosen D., Kazanietz M.G. (2012). Proteins kinase Cvarepsilon is required for non-small cell lung carcinoma growth and regulates the expression of apoptotic genes. Oncogene.

[56] Wang C., Ding C., Kong M., Dong A., Qian J., Jiang D., Shen Z. (2011). Tumor-targeting magnetic lipoplex delivery of short hairpin RNA suppresses IGF-1R overexpression of lung adenocarcinoma A549 cells *in vitro* and *in vivo*. Biochem. Biophys. Res. Commun..

[57] Vasudevan S., Tong Y., Steitz J.A. (2007). Switching from repression to activation: MicroRNAs can up-regulate translation. Science.

[58] Bruno I.G., Karam R., Huang L., Bhardwaj A., Lou C.H., Shum E.Y., Song H.W., Corbett M.A., Gifford W.D., Gecz J. (2011). Identification of a microRNA that activates gene expression by repressing nonsense-mediated RNA decay. Mol. Cell.

[59] Krutzfeldt J., Rajewsky N., Braich R., Rajeev K.G., Tuschl T., Manoharan M., Stoffel M. (2005). Silencing of microRNAs *in vivo* with “antagomirs”. Nature.

[60] Misso G., di Martino M.T., de Rosa G., Farooqi A.A., Lombardi A., Campani V., Zarone M.R., Gulla A., Tagliaferri P., Tassone P. (2014). MiR-34: A new weapon against cancer?. Mol. Ther. Nucleic Acids.

[61] Trang P., Wiggins J.F., Daige C.L., Cho C., Omotola M., Brown D., Weidhaas J.B., Bader A.G., Slack F.J. (2011). Systemic delivery of tumor suppressor microRNA mimics using a neutral lipid emulsion inhibits lung tumors in mice. Mol. Ther..

[62] Yanaihara N., Caplen N., Bowman E., Seike M., Kumamoto K., Yi M., Stephens R.M., Okamoto A., Yokota J., Tanaka T. (2006). Unique microRNA molecular profiles in lung cancer diagnosis and prognosis. Cancer Cell.

[63] Takamizawa J., Konishi H., Yanagisawa K., Tomida S., Osada H., Endoh H., Harano T., Yatabe Y., Nagino M., Nimura Y. (2004). Reduced expression of the *let-7* microRNAs in human lung cancers in association with shortened postoperative survival. Cancer Res..

[64] Johnson C.D., Esquela-Kerscher A., Stefani G., Byrom M., Kelnar K., Ovcharenko D., Wilson M., Wang X., Shelton J., Shingara J. (2007). The *let-7* microRNA represses cell proliferation pathways in human cells. Cancer Res..

[65] Kumar M.S., Erkeland S.J., Pester R.E., Chen C.Y., Ebert M.S., Sharp P.A., Jacks T. (2008). Suppression of non-small cell lung tumor development by the *let-7* microRNA family. Proc. Natl. Acad. Sci. USA.

[66] He X.Y., Chen J.X., Zhang Z., Li C.L., Peng Q.L., Peng H.M. (2010). The *let-7a* microRNA protects from growth of lung carcinoma by suppression of k-Ras and c-Myc in nude mice. J. Cancer Res. Clin. Oncol..

[67] Esquela-Kerscher A., Trang P., Wiggins J.F., Patrawala L., Cheng A., Ford L., Weidhaas J.B., Brown D., Bader A.G., Slack F.J. (2008). The *let-7* microRNA reduces tumor growth in mouse models of lung cancer. Cell Cycle.

[68] Trang P., Medina P.P., Wiggins J.F., Ruffino L., Kelnar K., Omotola M., Homer R., Brown D., Bader A.G., Weidhaas J.B. (2010). Regression of murine lung tumors by the *let-7* microRNA. Oncogene.

[69] Rai K., Takigawa N., Ito S., Kashihara H., Ichihara E., Yasuda T., Shimizu K., Tanimoto M., Kiura K. (2011). Liposomal delivery of microRNA-7-expressing plasmid overcomes epidermal growth factor receptor tyrosine kinase inhibitor-resistance in lung cancer cells. Mol. Cancer Ther..

[70] Wu Y., Crawford M., Mao Y., Lee R.J., Davis I.C., Elton T.S., Lee L.J., Nana-Sinkam S.P. (2013). Therapeutic delivery of microRNA-29b by cationic lipoplexes for lung cancer. Mol. Ther. Nucleic Acids.

[71] Wiggins J.F., Ruffino L., Kelnar K., Omotola M., Patrawala L., Brown D., Bader A.G. (2010). Development of a lung cancer therapeutic based on the tumor suppressor microRNA-34. Cancer Res..

[72] Chiou G.Y., Cherng J.Y., Hsu H.S., Wang M.L., Tsai C.M., Lu K.H., Chien Y., Hung S.C., Chen Y.W., Wong C.I. (2012). Cationic polyurethanes-short branch PEI-mediated delivery of mirR145 inhibited epithelial-mesenchymal transdifferentiation and cancer stem-like properties and in lung adenocarcinoma. J. Control. Release.

[73] Li Y.J., Zhang Y.X., Wang P.Y., Chi Y.L., Zhang C., Ma Y., Lv C.J., Xie S.Y. (2012). Regression of A549 lung cancer tumors by anti-miR-150 vector. Oncol. Rep..

[74] Cortez M.A., Valdecanas D., Zhang X., Zhan Y., Bhardwaj V., Calin G.A., Komaki R., Giri D.K., Quini C.C., Wolfe T. (2014). Therapeutic delivery of miR-200c enhances radiosensitivity in lung cancer. Mol. Ther..

[75] Fortunato O., Boeri M., Verri C., Moro M., Sozzi G. (2014). Therapeutic use of microRNAs in lung cancer. BioMed Res. Int..

[76] Burnett J.C., Rossi J.J. (2012). RNA-based therapeutics: Current progress and future prospects. Chem. Biol..

[77] Merkel O.M., Rubinstein I., Kissel T. (2014). siRNA delivery to the lung: What’s new?. Adv. Drug Deliv. Rev..

[78] Jackson A.L., Bartz S.R., Schelter J., Kobayashi S.V., Burchard J., Mao M., Li B., Cavet G., Linsley P.S. (2003). Expression profiling reveals off-target gene regulation by RNAi. Nat. Biotechnol..

[79] Bridge A.J., Pebernard S., Ducraux A., Nicoulaz A.L., Iggo R. (2003). Induction of an interferon response by RNAi vectors in mammalian cells. Nat. Genet..

[80] Thomas M., Lu J.J., Chen J., Klibanov A.M. (2007). Non-viral siRNA delivery to the lung. Adv. Drug Deliv. Rev..

[81] Ahn C.H., Chae S.Y., Bae Y.H., Kim S.W. (2004). Synthesis of biodegradable multi-block copolymers of poly(l-lysine) and poly(ethylene glycol) as a non-viral gene carrier. J. Control. Release.

[82] Bramson J.L., Bodner C.A., Graham R.W. (2000). Activation of host antitumoral responses by cationic lipid/DNA complexes. Cancer Gene Ther..

[83] Whitmore M., Li S., Huang L. (1999). LPD lipopolyplex initiates a potent cytokine response and inhibits tumor growth. Gene Ther..

[84] Hamasaki T., Suzuki H., Shirohzu H., Matsumoto T., D’Alessandro-Gabazza C.N., Gil-Bernabe P., Boveda-Ruiz D., Naito M., Kobayashi T., Toda M. (2012). Efficacy of a novel class of RNA interference therapeutic agents. PLoS One.

[85] Honma K., Iwao-Koizumi K., Takeshita F., Yamamoto Y., Yoshida T., Nishio K., Nagahara S., Kato K., Ochiya T. (2008). *RPN2* gene confers docetaxel resistance in breast cancer. Nat. Med..

[86] Fujita Y., Yagishita S., Takeshita F., Yamamoto Y., Kuwano K., Ochiya T. (2015). Prognostic and therapeutic impact of RPN2-mediated tumor malignancy in non-small-cell lung cancer. Oncotarget.

